# Mutation analysis of *PAX6* in inherited and sporadic aniridia from northeastern China

**Published:** 2012-06-27

**Authors:** Yang Kang, Ying Lin, Xue Li, Qiong Wu, Lei Huang, Qingjun Li, Qi Hu

**Affiliations:** 1 Department of Ophthalmology, First Affiliated Hospital of Harbin Medical University, Harbin, Heilongjiang, China; 2 State Key Laboratory of Ophthalmology, Zhongshan Ophthalmic Center, Sun Yat-sen University, Guangzhou, China

## Abstract

**Purpose:**

Haplo-insufficiency at the paired box gene 6 (*PAX6*) locus causes aniridia,which is characterized by iris hypoplasia and other anterior and posterior eye defects leading to poor vision. This study aimed to identify novel *PAX6* mutations that lead to familial and sporadic aniridia in northeastern China.

**Methods:**

Two aniridia patients from a family and a sporadic patient underwent full ophthalmologic examinations. Genomic DNA was isolated from the affected individuals, 5 noncarriers in the family and 100 healthy normal controls. The coding regions and the adjacent intronic sequence of *PAX6* were amplified by polymerase chain reaction (PCR) and direct bidirectional sequencing.

**Results:**

A nonsense mutation in exon 9 (c.718C>T) was identified in the patients but not in any other unaffected families. A C>T substitution at codon 240 converts an arginine codon (CGA) to a termination codon (TGA).The same mutation was detected in the sporadic patient by chance.

**Conclusions:**

A mutation in the *PAX6* gene was confirmed to be capable of causing the classic aniridia phenotype. This is the first report on the “hotspot” c.718C>T transition from northeastern Chinese families.

## Introduction

Aniridia (OMIM 106210) is a panocular and bilateral disease with a prevalence of 1 in 64,000 to 96,000 in the general population. In addition to the absence of the iris, other ocular disorders include corneal opacity, lens dislocation or cataracts, glaucoma, retina foveal dysplasia, and optic nerve hypoplasia, among others [1]. Approximately two thirds of all cases are familial, as they follow an autosomal dominant inheritance with variable expressivity, whereas the remaining one third is sporadic [2]. Aniridia can occur isolated or as part of the WAGR (Wilms tumor, aniridia, genitourinary disorders, and mental retardation) syndrome [1].

Various mutations in the paired box gene 6 (*PAX6*) gene are primarily responsible for aniridia [3,4]. Encoding a transcription factor,the highly conserved *PAX6* gene plays a major role in developmental processes in several organs, including the eye [5,6]. Human *PAX6* spans 22 kb on chromosome 11p13 and consists of 14 exons that encode 422 amino acids as transcriptional regulators. It has two DNA-binding domains, a bipartite paired domain (PD) and a paired-type home domain (HD), as well as a transactivation domain-rich proline, serine, and threonine (PST) at the COOH-terminal end. The PD and the HD, which are separated by a linker region (LNK), are the structural bases for the binding activity of the PAX6 protein [7,8]. The identification of a large number of mutations confirmed the role of *PAX6* in human eye disease,and the features of aniridia also reflect the wide expression of *PAX6* in the developing eye, including the neurectoderm, the surface ectoderm, and their derivatives. Up to now, more than 300 *PAX6* mutations have been identified in aniridic subjects worldwide [9-11], and mutations in the *PAX6* gene have been reported in patients with various ethnicities. In the present study, the same mutation (c.718 C>T) was described in the *PAX6* gene in one northeastern Chinese family and in one sporadic patient with aniridia.

## Methods

One family and one sporadic patient diagnosed with aniridia at the 1st affiliated hospital of Harbin Medical University were from Heilongjiang Province in northeastern China. Two aniridia patients from the family,five non-carrier family members, one sporadic patient and one hundred healthy controls (34.79±7.51 years old, 46 males) were recruited for the study. Experimental protocols were approved by the Institutional Review Board and complied with the tenets of the Declaration of Helsinki. The patients and relatives were evaluated by an ophthalmologist after a careful clinical ocular evaluation to exclude congenital anomalies other than aniridia, such as Axenfeld Rieger syndrome, iridocorneal endothelial syndromes, Peter’s anomaly, or sclerocornea. The possibility of the relatives of the sporadic patient having aniridia was excluded by full ophthalmologic examination. All patients underwent full ophthalmologic examination, including visual acuity, slit lamp biomicroscopy, and measurement of intraocular pressure (IOP) by applanation tonometry. Pentacam (Oculus, Wetzlar, Germany) was used to assess the cornea and anterior segment. Fundus photograph was taken with the use of Heidelberg retina angiograph (Heidelberg Engineering, Heidelberg, Germany). Ophthalmoscope and eye-ground photography were used to estimate the retina and the optic nerve. Systemic examinations were performed to exclude associated anomalies (e.g., Wilms’ tumor, urogenital anomalies, and mental retardation) in all subjects included in this study.

### Molecular methods

Venous blood samples from 3 aniridia patients, 5 noncarriers in the family, and 100 healthy normal controls were collected for genomic DNA drawn from peripheral blood leucocytes with the use of standard protocols. Total genomic DNA was extracted from peripheral blood with the use of the QIAmp Blood kit (Qiagen, Hilden, Germany). Eleven coding exons (exon 4 to exon 13 and an extra exon, 5a) and the flanking regions of *PAX6* were amplified by polymerase chain reaction (PCR) with eight pairs of primers (Table 1) [12]. The DNA was subsequently purified with the use of the help of the Qiaex II kit (Qiagen). The purified DNA was sequenced with ABI BigDye Terminator Cycle Sequencing kit v3.1, (ABI Applied Biosystems, Foster City, CA); the sequencing results were bidirectional and compared with the reference sequences in the database at the National Center for Biotechnology Information (NCBI; NC_000011.9). Mutation descriptions follow the new nomenclature system recommended by the Human Genomic Variation Society (HGVS) [13]. Any detected variation in *PAX6* was further evaluated in 7 family members,in 1 sporadic patient, and in 100 normal controls with the use of heteroduplex PCR-single strand conformational polymorphism (SSCP) analysis as previously described in the literature [14]. One additional pair of primers for heteroduplex PCR-SSCP analysis [15] was synthesized for the amplification of exon 9 (Table 1).

**Table 1:** Primers used for PCR.

**Exon**	**Forward (5′-3′)**	**Reverse (5′-3′)**	**Product size (bp)**	**Annealing temperature (°C)**
4	GGACTTAGGGTTTGATGACAG	CCAGAAAGACCAGAGGCAC	684	50
5	GAGATTGGCACAGGTTGG	CATAAGTAGCATCGTTTACAGT	423	60
5 a,6	AACGCCACTTTAAGCAAGGT	GGAGGGCAGATGTTCTCAA	620	52
7	AATCCACCCACTGTCCCG	CCAGCCACCTTCATACCG	542	60
8	TCAGGTAACTAACATCGCA	GTTGACTGTACTTGGAAGAA	719	53
9,10,11	GAGGTGGGAACCAGTTTGATG	CAAGCCAATCTCTGTAGTGCG	890	52
12	GAGGCTTGATACATAGGC	CCATAAGACCAGGAGATT	452	58
13(1)	GTTTCTGAGGGTGCTACT	TTGAATGGCTAACTGGG	1519	48
13(2)	CAGTTTCTGAAGGTGCTA	TCCATCCAGTCTACATTG	578	48
9	GTAGTTCTGGCACAATATGG	GAGGTGCTTGTACAGAGTAC	83	55

Sequence of oligonucleotides and annealing temperatures used for exon-by-exon *PAX6* amplification.

## Results

### Clinical findings

**Figure 1:**
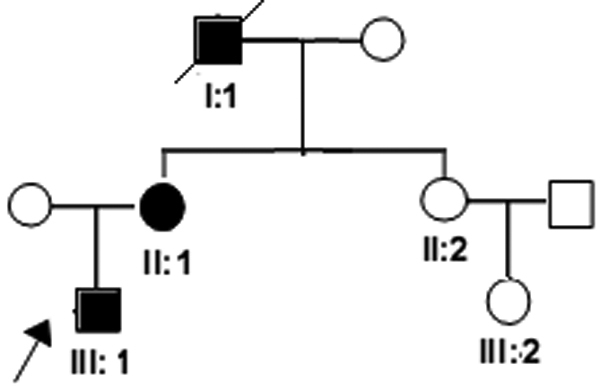
Pedigree of the family. Males and females are represented by squares and circles, respectively, and affected family members as confirmed by an ophthalmologist are represented by darkened symbols. The arrow points to the proband. The inheritance pattern in the family appears to be autosomal dominant.

**Figure 2:**
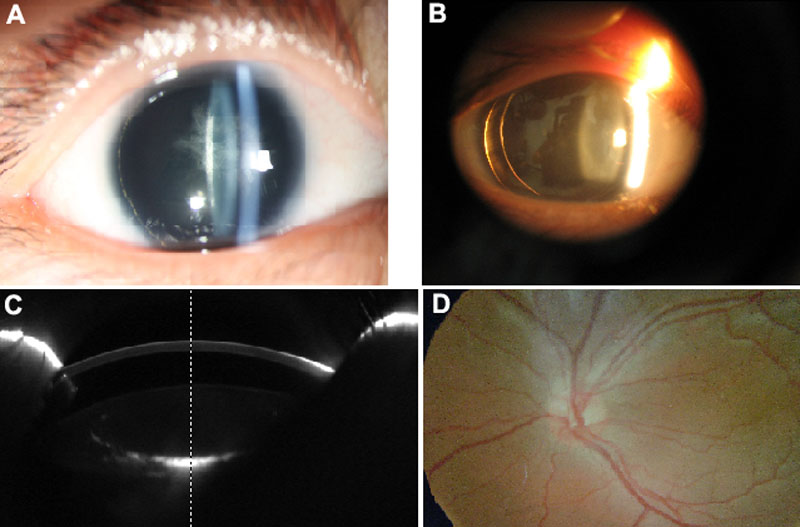
Photographic demonstration of aniridia expression in family members. The eyes of patient II:1 exhibit iris hypoplasia and lenses opacity (**A**), whereas those of patient III:1 had aniridia and transparent lenses (**B**). Pentacam photo shows the normal anterior segment picture of the patient II:1 from the family (**C**), and that of patient III:1 shows the same. The eyes of the family exhibited foveal hypoplasia by eyeground photography, including II:1 and III:1 (**D**).

**Figure 3:**
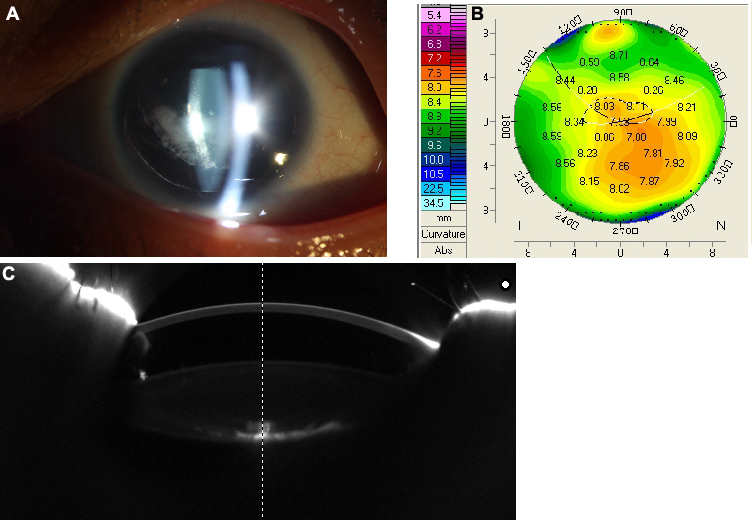
Photographic demonstration of aniridia expression in the sporadic patient. The sporadic patient suffered complete bilateral defects of the iris and a cataract (**A**). Pentacam photo shows that the cornea of the sporadic patient is irregular (**B**). The anterior segment is shorter than normal (**C**), as measured by Pentacam.

Three individuals in three successive generations (Figure 1) were found to have similar congenital ocular disease. In the family, two living patients had bilateral aniridia and horizontal tremor, and they had subnormal vision even from early childhood. Another similar phenotype is characterized by clear and normal-sized corneas and anterior chamber (Figure 2C) by Pentacam photo, and normal IOP and foveal hypoplasia with absence of foveolar and perifoveal reflexes were observed (Figure 2D) in both eyes. No other abnormalities were found in the retina, choroids, or optic nerve head in the family. Patient II:1 in the family had a congenital cataract (Figure 2A), whereas no abnormalities were detected in the lens in patient III:1 (Figure 2B). The sporadic patient is a female aged 30 years old. The defining characteristics of the patient were subnormal vision nystagmus, irregular cornea (Figure 3B) as shown by Pentacam, bilateral aniridia, and congenital cataract (Figure 3A). The anterior chamber depth for the sporadic patient was 1.87 mm (OD) and 1.96 mm (OS), which is shorter than normal during her first evaluation (Figure 3C). She demonstrated an IOP of approximately 28 mmHg and incipient glaucomatous changes in the optic cup in both eye.

### Mutation screening

**Figure 4:**
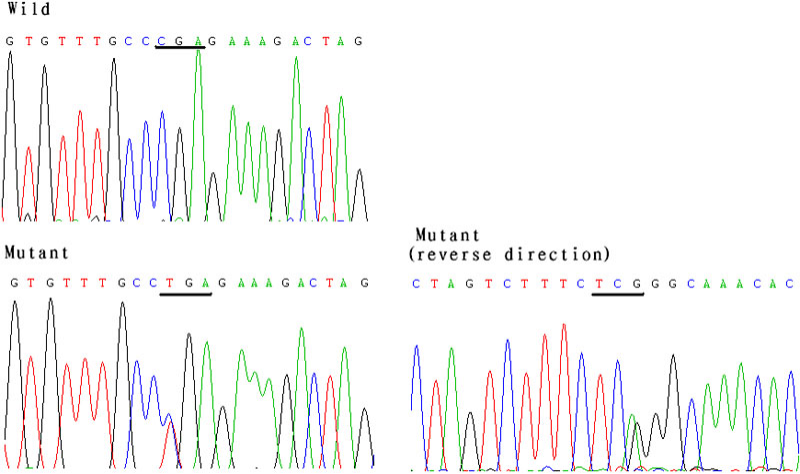
Nonsense mutation (c.718C>T) in the families and in the sporadic patient in northeastern China. DNA sequence of a part of *PAX6* in the affected patients and unaffected individuals. The wild shows the corresponding normal sequence from the unaffected families and the normal controls. The mutant presents mutation sequence in two patients of the family and in the sporadic patient as confirmed by sense and antisense strands. It is a nonsense mutation(c.718 C>T) in exon 9.

The family exhibited a nonsense mutation (Figure 4) in exon 9 (c.718 C>T) but not in any other coding exons in *PAX6*. Mutations were confirmed by sequencing both sense and antisense strands in the patients and in the unaffected families. The same mutation c.718 C>T in exon 9 was unexpectedly detected in the sporadic patient. C>T substitution at codon 240 converts an arginine codon (CGA) to a termination codon (TGA), which is R240X. The mutation in three patients was also detected by SSCP analysis. No mutations were detected in any of the unaffected family members or control samples. This study is the first to report on c.718 C>T transition in northeastern China.

## Discussion

Aniridia is a human eye malformation caused by heterozygous null mutations of *PAX6*, which is extraordinarily conserved throughout evolution. The molecular bases for aniridia are derived from mutations of *PAX6* that lead to premature protein termination [16]. It is assumed to cause loss of function of one allele and thus result in 50% reduction in overall activity rather than accumulation of dominant or dominant-negative forms of the protein [17]. Therefore, haplo-insufficiency of the *PAX6* gene has been suggested to underlie the aniridia phenotype.

In this study, a mutation (c.718 C>T, p.R240X) in a Chinese family and in a sporadic patient with aniridia, which is not a polymorphism in the normal population was described. The nonsense mutation in exon 9 has been reported as a mutation hotspot for *PAX*6 in other ethnic pedigrees. Only one report in a Chinese family from Zhongshan Ophthalmic Center in south China existed [18]. However, it is the first mutation reported in northeastern Chinese patients. From the family’s data, we could determine that the affected members from the same family with the same mutation cause different phenotypes. Patient II:1 had a congenital cataract, whereas no abnormalities were detected in the lens in patient III:1. Patient II: 1in the family and the sporadic patient were found to have similar phenotypes in lens. Therefore, *PAX6* plays a pleiotropic role in variable phenotypes among individuals [19]. The reason for this varying phenotype among individuals with the same mutation is worth exploring [20]. Some hypomorphic alleles with reduced activity probably generate less severe or variant phenotypes [21]. In such case, nonsense mutations are deemed to be mostly related to variant phenotypes,a result indicating that the altered function of the protein may trigger distinct or milder phenotypes compared with complete loss of function. c.718 C>T in exon 9 has been previously reported several times in other ethnic groups and appears to represent a hotspot of *PAX6* mutation, the mutation was predicted to be attributed to methylated cytosine deamination in *PAX6* [20].

In summary, this study identified one mutation of *PAX6* first reported in northeastern Chinese patients with aniridia. Our genetic analysis provides further examples of haplo-insufficiency of *PAX*6 in aniridia. It is also valuable for genetic counseling and prenatal diagnosis in families where aniridia appears.
